# Psychometric properties of a Korean version of the pre-sleep arousal scale

**DOI:** 10.1371/journal.pone.0333390

**Published:** 2025-09-26

**Authors:** Namhee Kim, Bo Gyeong Lee

**Affiliations:** 1 Department of Nursing, Hanseo University, Seosan-Si, Republic of Korea; 2 College of Nursing, Daegu Catholic University, Daegu, Republic of Korea; Fakultet za pravne i poslovne studije dr Lazar Vrkatic, SERBIA

## Abstract

**Purpose:**

Sleep quality is a multidimensional construct encompassing the effectiveness and restorativeness of sleep. The pre-sleep arousal scale is a widely used instrument for evaluating aspects of arousal that are closely related to sleep quality. This study aimed to evaluate the psychometric properties of the Korean version of the pre-sleep arousal scale (K-PSAS).

**Methods:**

We performed a secondary analysis of cross-sectional data from 286 adults aged 19–70 years who used electronic cigarettes or heated tobacco products. The original PSAS was translated into Korean, with content validity assessed by experts. Construct validity was evaluated via exploratory factor analysis, and concurrent validity was assessed by correlating the K-PSAS with the Insomnia Severity Index, Pittsburgh Sleep Quality Index, and Hospital Anxiety and Depression Scale. Reliability was examined using Cronbach’s α, and split-half reliability coefficient.

**Results:**

Both the item-level content validity index for all items and the scale-level content validity index average for the K-PSAS-16 were 1.0. After removing the survey item on “being mentally alert and active at bedtime” (item 13) due to low factor loading, the K-PSAS-15 demonstrated a two-factor structure, with somatic and cognitive arousal factors explaining 42.36% and 10.19% of the variance, respectively. A significant positive correlation was observed between the two factors (ρ = 0.61, *p* < .001). Item 13 also showed a low corrected item-total correlation coefficient of −0.10. The correlation coefficients between K-PSAS-15 and other validated scales ranged from 0.49 to 0.71. The K-PSAS-15 showed good internal consistency, with a Cronbach’s α of.91 for the total scale, and α = .87 and.90 for the somatic and cognitive subscales, respectively. Split-half reliability was also acceptable (total = .90; somatic = .86; cognitive = .88).

**Conclusion:**

The K-PSAS-15, which excludes one poorly performing item from the original scale, is a reliable and valid tool for assessing pre-sleep arousal.

## Introduction

Insomnia disorders, which are the most common sleep disorders, are defined as difficulty initiating or maintaining sleep [1,2]. Many previous studies have shown that insomnia has negative impacts on various aspects of physical and mental health, including fatigue, depression, and cardiovascular disease, all with the ultimate effect of reducing quality of life [1–3]. The prevalence of insomnia varies depending on the diagnostic tools used along with the demographic factors of age and gender [1,2]. However, in general, about 10–25% of adults report subjective sleep dissatisfaction, while 30–36% of adults report difficulty in initiating or maintaining sleep at night [1,2].

Hyperarousal refers to increased levels of cognitive, affective, physiological, and physical activation, or a diminished capacity to attenuate such arousal. It constitutes a core component of insomnia disorder [4–6]. Increased arousal before sleep can lead to hyperactivation of various sensory and information-processing systems, therefore making it difficult to fall asleep and reducing sleep quality [6]. Many studies have classified pre-sleep arousals into cognitive and somatic categories [7–10]. Somatic arousal is characterized by physiological symptoms such as a rapid or irregular heartbeat, shortness of breath, and muscle tension [7]. In contrast, cognitive arousal includes symptoms such as worry about falling asleep, racing thoughts, and feelings of anxiety or depression [7]. Numerous studies have demonstrated a significant association between increased cognitive arousal before sleep and insomnia. Conversely, increased somatic arousal has been observed in only a limited subset of patients with chronic insomnia [4–6,10]. Ultimately, both cognitive and somatic pre-sleep arousal contribute to difficulties with sleep onset and reduced overall sleep quality.

Commonly used self-report instruments to measure sleep quality include the Insomnia Severity Index (ISI) [8], Pittsburgh Sleep Quality Index (PSQI) [9], and Pre-Sleep Arousal Scale (PSAS) [7]. The PSAS was developed to determine the extent to which excessive psychophysiological arousal before sleep increases sleep latency [7]. Nicassio and colleagues developed 16 items, consisting of eight items measuring each of somatic and cognitive arousals, to measure pre-sleep arousal [7]. The developed PSAS showed significant correlations with general anxiety, depression, and other self-reported sleep indices [7]. This instrument also showed a high correlation with delayed sleep onset, decreased total sleep time, and maintaining uninterrupted sleep [7]. The PSAS was also tested for discriminant validity, and there was a significant difference in sleep latency between the normal sleep group and the insomnia group [7].

The PSAS has been translated into several languages, and there have been multiple studies evaluating its cross-cultural psychometric properties. One such study examined the psychometric properties of the PSAS in a large community sample in Sweden [11]. In particular, Jansson-Fröjmark and colleagues [11] evaluated a Swedish version of PSAS-13 by removing items 9, 11, and 16 from the cognitive subscale, as those items had low factor loading values. The Sweden version of PSAS-13 had two subscales, and factorial, discriminant, and convergent validities were all established [11]. Another study examined the validity of a Japanese version of the PSAS (PSAS-J). The PSAS-J has good internal reliability and test-retest reliability, along with excellent concurrent validity for anxiety and depression [12]. Several prior studies have shown that the PSAS is a valuable instrument for assessing pre-sleep arousal. Nevertheless, the psychometric properties of the Korean version of the PSAS (K-PSAS) have yet to be established. Therefore, in the present study, we evaluated the cross-cultural validity of the K-PSAS among Korean adults.

To date, the PSAS has been utilized to assess sleep quality in pregnant women, patients with insomnia, psoriasis, and depression, as well as in the general population [13–15]. However, the psychometric properties of the PSAS have not been systematically evaluated in smokers. Validity refers to the degree to which test scores reflect the characteristics of the population from which they are derived [16]. Therefore, establishing validity separately for specific populations is essential to avoid biased conclusions regarding the latent traits measured by research instruments [16]. Nicotine in cigarettes is a representative substance that stimulates nerve transmission and induces cognitive arousal [17]. Assessing the level of pre-sleep arousal may be a meaningful predictor of sleep quality among smokers. Given the increasing prevalence of electronic cigarettes (e-cigarettes) and heated tobacco products (HTPs) use in South Korea, it is crucial to ensure that psychometric tools such as the PSAS are valid and reliable within this specific population. Because hyperarousal is a core mechanism of insomnia, the validation of reliable tools such as the PSAS is essential for accurately assessing this construct. Accordingly, this study aimed to evaluate the psychometric properties of a K-PSAS and to examine its applicability to smokers, especially e-cigarette or HTP users.

## Materials and methods

### Study design and participants

We performed a secondary analysis of existing data that had been collected to examine sleep quality in e-cigarette or HTP users. In the primary study, an online survey was conducted with participants consisting of adults aged from 19 to 70 years old who used e-cigarettes or HTPs. The exclusion criteria in that study comprised those who were previously diagnosed with or currently had a sleep-related disorder (e.g., obstructive sleep apnea syndrome, restless legs syndrome, and periodic limb movement disorder), night workers, shift workers, and those taking medications related to other mental disorders. The final sample included adults with varied sociodemographic characteristics—such as age, gender, education, and employment status—which are summarized in Table 1. In the primary study, 286 participants were ultimately recruited through convenience sampling, thus meeting the requirement of 10 subjects per item for exploratory factor analysis [18].

**Table 1:** General characteristics of participants (N = 286).

Variables/ Categories	n	%
Age (years)		
≤ 30	69	24.1
31-50	189	66.1
≥ 51	28	9.8
Gender		
Male	245	85.7
Female	41	14.3
Education level		
≤ High school	60	21.0
≥ College	226	79.0
Marital status		
Without partner	123	43.0
With partner	163	57.0
Employment status		
Unemployed	29	10.1
Employed	257	89.9
Household income (n = 265)		
Low	120	45.3
High	145	54.7
Body Mass Index (kg/m^2^)		
≤ 22.9	77	27.1
23.0-24.9	72	25.4
≥ 25.0	135	47.5
Health status^a^		
Good	114	39.9
Moderate	136	47.6
Bad	36	12.6
Perceived stress level^a^		
Low	124	43.4
Moderate	100	35.0
High	62	21.7

K-PSAS = Korean version of the Pre-Sleep Arousal Scale.

^a^Not total 100% due to rounding.

### Instruments

#### Pre-sleep arousal scale (PSAS).

The PSAS was developed to assess pre-sleep arousal by Nicassio et al. (1985) [7]. The PSAS is a self-report questionnaire that consists of 16 items in total, including eight items for somatic arousal (i.e., heart racing, nervous feeling, difficulty breathing, muscle stiffness, cold feeling, uncomfortable feeling in the stomach, perspiration, and dry feeling in mouth or throat) and eight items for cognitive arousal (i.e., worry about falling asleep, review or ponder events of the day, depressing or anxious thoughts, worry about problems other than sleep, being mentally alert. thoughts that cannot be shut out, thoughts keep racing though my head, and being distracted by sounds, noise in the environment), regarding the level of arousal at bedtime [7]. Participants were asked to respond on a 5-point scale from 1 (not at all) to 5 (extremely) regarding the severity of their pre-sleep arousal symptoms, with higher scores indicating greater pre-sleep arousal.

#### Insomnia severity index (ISI).

The Insomnia Severity Index (ISI) was developed by Bastien et al. (2001) to assess the severity of insomnia symptoms and sleep quality in individuals with insomnia [8]. Participants respond to a total of 7 items about their sleep status, each rated on a 5-point Likert scale ranging from 0 (no problem) to 4 (very severe problem) [8]. The total score on this item ranges from 0 to 28, with higher scores indicating poorer sleep quality. The ISI total score cutoff criteria are as follows: 0–7, no clinical symptoms of insomnia; 8–14, potential insomnia; 15–21, moderate insomnia; 22–28, severe insomnia [8,19]. ISI has been translated into several languages, and its validity has been established [20,21]. Regarding the Korean version, good internal consistency has been reported, with a Cronbach’s alpha of 0.92 [19]. The Cronbach’s alpha in the present study was 0.84.

#### Pittsburgh sleep quality index (PSQI).

The PSQI is a 19-item self-report questionnaire developed by Buysse et al.(1989) to evaluate sleep quality [9]. It comprises seven components (i.e., subjective sleep quality, sleep onset latency, sleep duration, sleep efficiency, sleep disturbance, use of sleep medication, and daytime dysfunction), each of which is scored from 0 (no difficulty) to 3 (severe difficulty) points. The total score ranges from 0 to 21, with higher mean scores representing poorer sleep quality. Moreover, a mean score greater than 5 is considered indicative of poor sleep quality [9]. The PSQI has been translated into and validated in many languages [22,23], and the reliability and validity of the Korean version of the PSQI have already been established [24]. The Cronbach’s alpha in the current study was 0.73.

#### Hospital anxiety and depression scale (HADS).

The HADS was designed by Zigmond and Snaith (1983) to assess participants’ experiences of emotions over the past week [25]. The HADS is a widely used instrument that assesses anxiety and depression, and it has also been validated for evaluating sleep quality in both hospital patients and the general population [11,25,26]. It is divided into subscales of anxiety and depression, where each subscale consists of 7 items (HADS-A and HADS-D) [25]. This instrument is answered on a 4-point scale from 0 (not at all) to 3 (most of the time). Each subscale is scored from 0 to 21, with higher scores indicating higher levels of anxiety or depression. The Cronbach’s alpha values of the Korean versions of HADS-A and HADS-D have been shown to be 0.89 and 0.86, respectively [27]. The Cronbach’s alpha values of HADS-A and HADS-D in this study were shown to be 0.83 and 0.73, respectively.

### Procedure

Primary data collection was conducted from March to October 2023. In screening potential participants, four health managers from one metropolitan city and one province were recruited. Researchers explained the study to participants and provided a link to the online survey via mobile phone only to those who had voluntarily agreed to participate. To prevent the collection of insincere responses during the online survey, participants were asked to answer all questions except for smoking-related questions. Original data were collected after approval from the researcher’s university Institutional Review Board (IRB) (ref no. CUIRB-2022–0067). Participants were informed that their data could be shared for research purposes and written consent was obtained. All participant information in the original data is recorded anonymously, making it impossible to identify individuals. This secondary data analysis study was approved by the same IRB as exempt from review (ref no. CUIRB-2024-E001).The PSAS was translated into Korean following cross-cultural adaptation procedures for self-report measures [28]. Two researchers with doctoral degrees in nursing translated the PSAS into Korean; the two different translations were agreed upon with any discrepancies reconciled through discussion. It was then back-translated into English by two bilingual experts. For translations that were inconsistent with the original PSAS, a consensus was reached within the expert group by considering cultural and linguistic aspects. The preliminary K-PSAS was pilot-tested on five e-cigarette or HTP users. Afterward, the results were revised and supplemented, at which point the translation of the K-PSAS was considered to be completed. All measurement tools used in this study were approved by their developers or the authorities.

### Data analysis

All analyses were performed using IBM SPSS Statistics version 25.0 (IBM Corp., Armonk, NY, USA). The general characteristics of the participants were analyzed using descriptive statistics (n, %). The psychometric properties of K-PSAS were confirmed through content validity, construct validity, item analysis, concurrent validity, and reliability.

Item-level content validity index (I-CVIs) and the scale-level content validity index/average (S-CVI/Ave) for scale were assessed by four experts, including two sleep consultants and two nursing professors. Polit and Beck recommended an I-CVIs of 1.0 and an S-CVI/Ave of 0.9 or higher from 3 to 4 experts [29]. [30]. Then, the principal axis factoring with oblimin oblique was employed. The number of K-PSAS factors was determined using the Guttman-Kaiser criterion (eigenvalues ≥ 1.0) and the Cattell scree test [31]. Factor loadings greater than 0.4 were the cutoff for item retention [31].

In the item analysis, the mean and standard deviation, corrected item-total correlation coefficients, and Cronbach’s alpha if item deleted were evaluated. The Spearman’s rho correlation coefficient was calculated to determine the correlation between K-PSAS and ISI, PSQI, HADS-A, and HADS-D.

Cronbach’s α was evaluated to assess internal reliability, with values between 0.70 and 0.79 considered acceptable and values above 0.80 regarded as good [32]. Additionally, split-half reliability was calculated using the Spearman–Brown coefficient to evaluate the consistency between two halves of the scale, providing further evidence of internal coherence.

## Results

### General characteristics of participants

A total of 286 participants were included. Most were male (85.7%), aged 31–50 years (66.1%), college-educated or higher (79.0%), employed (89.9%), and living with a partner (57.0%). Among 265 respondents, 54.7% reported high household income. Regarding BMI, 47.5% were classified as ≥25.0 kg/m². Health status was rated as moderate by 47.6% and good by 39.9%. Perceived stress was low in 43.4% and high in 21.7% (Table 1).

### Content validity

The content validity of the K-PSAS-16 was supported by I-CVI values of 1.0 for all items and an S-CVI/Ave of 1.0.

### Factorial construct validity

**Fig 1:**
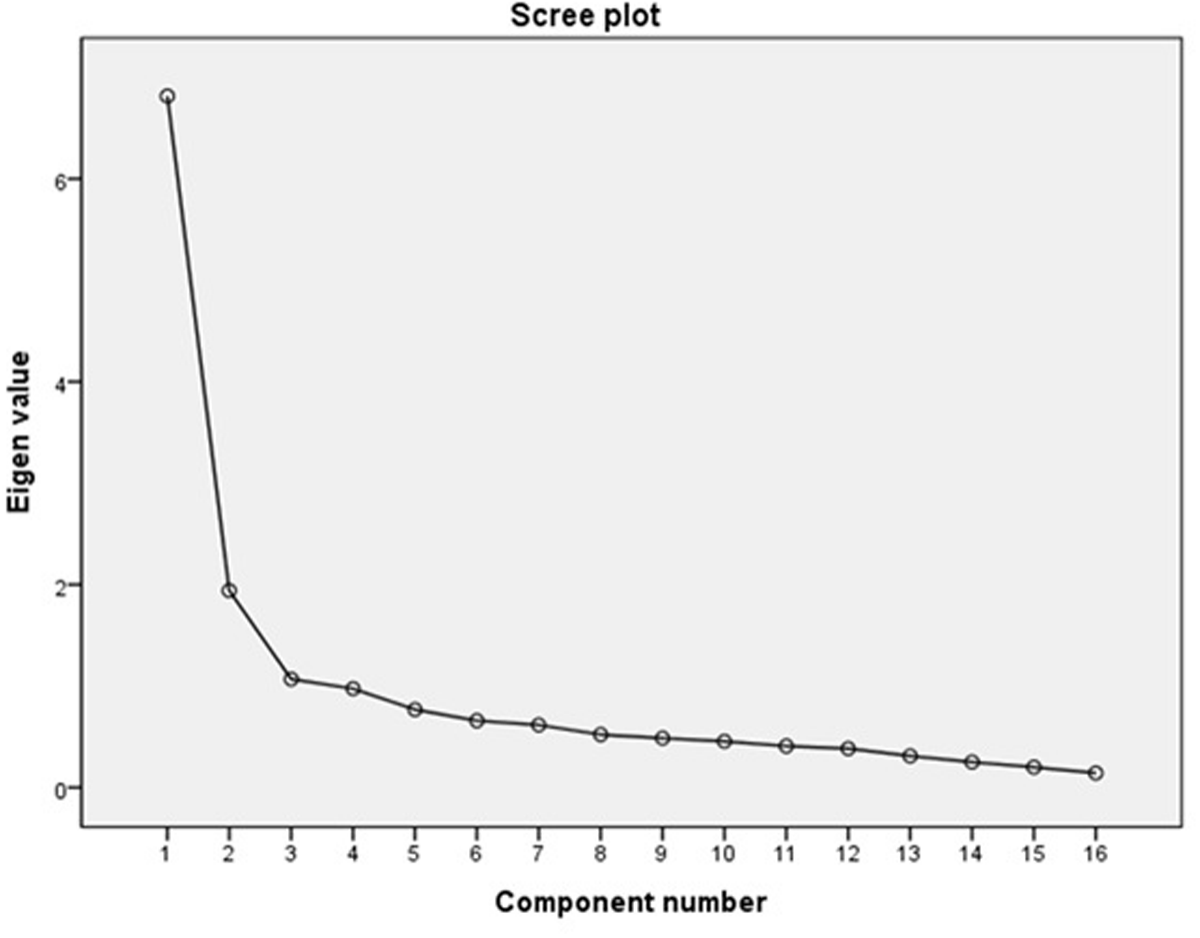
Scree plot of K-PSAS-15.

As a result of conducting EFA of K-PSAS-16, the KMO index was found to be 0.90 and the Bartlett test of sphericity was found to be significant (*p* < .001). EFA of the initial K-PSAS-16 revealed a two-factor structure consistent with the original PSAS. The two factors, somatic arousal and cognitive arousal, explained 39.78% and 9.56% of the total variance, respectively. The factor loadings of somatic arousal excluding item 13 (“being mentally alert, active”) were 0.47 to 0.83, while the factor loadings of cognitive arousal were −0.51 to −0.92. The factor loadings of item 13 were −0.10 and 0.02 for somatic arousal and cognitive arousal, respectively. After removing item 13, secondary EFA was conducted on the K-PSAS-15, which consists of the remaining 15 items. The scree plot indicated that the K-PSAS-15 also followed a two-factor structure (Fig 1).

Principal axis factoring was used on data from 286 participants. Two factors were retained based on the Guttman-Kaiser criterion (eigenvalues > 1.0) and visual inspection of the inflection point in the scree plot. In K-PSAS-15, the factor loadings were found to be 0.47 to 0.83 for somatic arousal and −0.52 to −0.92 for cognitive arousal. The two-factor structure of the K-PSAS-15 explained 42.36% and 10.19% of the total variance through the cognitive and somatic arousal factors, respectively. In the K-PSAS-15, a positive correlation was observed between somatic and cognitive arousal (ρ = 0.61, *p* < .001) (Table 2).

**Table 2:** Results of exploratory factor analysis for the K-PSAS-16 and K-PSAS-15 (N = 286).

Items	Factor loadings with K-PSAS-16		Factor loadings with K-PSAS-15	
	Somatic arousal	Cognitive arousal	Somatic arousal	Cognitive arousal
Somatic Q3	0.83	0.18	0.83	0.17
Somatic Q1	0.81	0.05	0.80	0.03
Somatic Q4	0.73	0.03	0.73	0.02
Somatic Q2	0.69	−0.13	0.68	−0.14
Somatic Q6	0.66	−0.08	0.65	−0.09
Somatic Q5	0.60	−0.04	0.60	−0.05
Somatic Q8	0.50	−0.18	0.49	−0.18
Somatic Q7	0.47	−0.05	0.47	−0.05
Cognitive Q15	−0.09	−0.92	−0.10	−0.92
Cognitive Q14	−0.14	−0.91	−0.14	−0.91
Cognitive Q10	0.01	−0.78	0.01	−0.78
Cognitive Q12	0.12	−0.73	0.12	−0.74
Cognitive Q11	0.31	−0.54	0.30	−0.55
Cognitive Q9	0.13	−0.53	0.12	−0.53
Cognitive Q16	0.18	−0.51	0.18	−0.52
Cognitive Q13	−0.10	0.02	Deleted	Deleted
Eigen value	5.34	5.28	5.27	5.29
Explained variance (%)	39.78	9.56	42.36	10.19
	KMO = 0.90, Bartlett’s χ^2 ^= 2,415.82 (*p *< .001)		KMO = 0.90, Bartlett’s χ^2 ^= 2,407.15 (*p *< .001)	
**Factor correlation (ρ, *p*)**			**Somatic** **arousal**	**Cognitive arousal**
Somatic arousal			1.00	0.61 (<.001)
Cognitive arousal				1.00

Note. Factor extraction was conducted using principal axis factoring with oblimin oblique rotation.

The number of factors was determined using the Guttman-Kaiser criterion (eigenvalues ≥ 1.0) and Cattell’s scree test. Factor loadings ≥ 0.40 were considered sufficient for item retention.

K-PSAS = Korean version of the Pre-Sleep Arousal Scale; KMO = Kaiser-Meyer-Olkin.

### Item statistics

The mean total score of the K-PSAS-16 was 27.52 ± 9.20. The mean scores of the somatic arousal and cognitive arousal subscales were 11.70 ± 4.62 and 15.82 ± 5.77, respectively. For the somatic arousal subscale, item mean scores ranged from 1.29 ± 0.71 to 1.94 ± 1.02, with the lowest for item 5 (“cold feeling in your hands, feet or your body in general”) and the highest for item 8 (“dry feeling in mouth or throat”). In the cognitive arousal subscale, item mean scores ranged from 1.49 ± 0.88 to 2.54 ± 1.06, with the lowest for item 9 (“worry about falling asleep”) and the highest for item 13 (“being mentally alert, active”). The corrected item-total correlation coefficients—excluding item 13—ranged from 0.43 to 0.72, with most items exceeding 0.30. Cronbach’s α if item were deleted ranged from 0.88 to 0.91. In comparison, the mean total score of the K-PSAS-15 was 23.53 ± 8.84, while the mean score of the cognitive arousal subscale, excluding item 13, was 13.28 ± 5.76 (Table 3).

**Table 3:** Item statistics for the K-PSAS-16 and K-PSAS-15 (N = 286).

Subscales	Items	Mean ± SD	Corrected item-total correlation	Cronbach’s α if item deleted
Somatic arousal	Q1. Heart racing, pounding or beating irregularly	1.42 ± 0.78	0.60	0.88
	Q2. A jittery, nervous feeling in your body	1.45 ± 0.79	0.67	0.88
	Q3. Shortness of breath or labored breathing	1.30 ± 0.69	0.50	0.89
	Q4. A tight, tense feeling in your muscles	1.33 ± 0.70	0.56	0.89
	Q5. Cold feeling in your hands, feet or your body in general	1.29 ± 0.71	0.54	0.89
	Q6. Have stomach upset	1.52 ± 0.84	0.61	0.88
	Q7. Perspiration in palms of your hands or other parts of your body	1.44 ± 0.86	0.43	0.89
	Q8. Dry feeling in mouth or throat	1.94 ± 1.02	0.56	0.88
	Somatic arousal subtotal	11.70 ± 4.62		
Cognitive arousal	Q9. Worry about falling asleep	1.49 ± 0.88	0.55	0.89
	Q10. Review or ponder events of the day	2.09 ± 1.09	0.67	0.88
	Q11. Depressing or anxious thoughts	1.67 ± 0.98	0.71	0.88
	Q12. Worry about problems other than sleep	2.04 ± 1.05	0.72	0.88
	Q13. Being mentally alert, active	2.54 ± 1.06	−0.10	0.91
	Q14. Can’t shut off your thoughts	2.06 ± 1.13	0.65	0.88
	Q15. Thoughts keep running through your head	2.11 ± 1.11	0.69	0.88
	Q16. Being distracted by sounds, noise in the environment	1.82 ± 1.05	0.60	0.88
	Cognitive arousal subtotal (8 items)	15.82 ± 5.77		
	Cognitive arousal subtotal (excluding item 13)	13.28 ± 5.76		
K-PSAS-16	Total	27.52 ± 9.20		
K-PSAS-15	Total (excluding item 13)	23.53 ± 8.84		

K-PSAS = Korean version of the Pre-Sleep Arousal Scale.

### Concurrent validity

The K-PSAS-16 showed significant correlations with the ISI (r = .55), PSQI (r = .57), HADS-A (r = .69), and HADS-D (r = .46). The K-PSAS-15 also showed significant correlations with the ISI (r = .58), PSQI (r = .61), HADS-A (r = .71), and HADS-D (r = .49). For the subscales of the K-PSAS-15, the somatic arousal subscale correlated with the ISI (r = .38), PSQI (r = .42), HADS-A (r = .53), and HADS-D (r = .38), while the cognitive arousal subscale correlated with the ISI (r = .60), PSQI (r = .62), HADS-A (r = .71), and HADS-D (r = .47). All correlations were statistically significant at *p* < .001. (Table 4).

**Table 4:** Correlation coefficients between the K-PSAS, ISI, PSQI, and HADS (N = 286).

Variables	ISI	PSQI	HADS-A	HADS-D
	ρ (*p*)			
K-PSAS-16	0.55 (<.001)	0.57 (<.001)	0.69 (<.001)	0.46 (<.001)
K-PSAS-15	0.58 (<.001)	0.61 (<.001)	0.71 (<.001)	0.49 (<.001)
K-PSAS-somatic (8 items)	0.38 (<.001)	0.42 (<.001)	0.53 (<.001)	0.38 (<.001)
K-PSAS-cognitive (7 items)	0.60 (<.001)	0.62 (<.001)	0.71 (<.001)	0.47 (<.001)

Note. All coefficients represent Spearman’s rank-order correlations (ρ) with associated p-values.

K-PSAS = Korean version of the Pre-Sleep Arousal Scale; ISI = Insomnia Severity Index; PSQI = Pittsburgh Sleep Quality Index; HADS-A = Hospital Anxiety and Depression Scale Anxiety; HADS-D = HADS Depression.

### Reliability

The K-PSAS-15 demonstrated excellent internal consistency, with a Cronbach’s α of.91 for the total scale. Both subscales also showed high internal consistency, with α = .87 for the somatic arousal subscale and α = .90 for the cognitive arousal subscale. The odd-even split-half reliability coefficient was.90 for the K-PSAS-15, with coefficients of.86 and.88 for the somatic and cognitive arousal subscales, respectively.

## Discussion

In this study, the psychometric properties of the Korean version of the PSAS, which assesses pre-sleep arousal as a key indicator of sleep quality, were evaluated among users of e-cigarettes and HTPs. The K-PSAS was divided into two subscales consistent with the division of the original PSAS. The results are consistent with the findings of previous studies that translated PSAS into several languages. The Swedish, Japanese, Turkish, and Portuguese versions of the PSAS showed that a two-factor model consisting of somatic and cognitive arousal was appropriate, despite the possible variability in the numbers and ages of the study population [11,12,33,34]. However, in the Portuguese version of the PSAS, the somatic factor was divided into two factors, so three factors also seemed plausible [34]. Even though there are some differences between the different versions, it has clearly been shown in a few studies that, in general, PSAS is a two-factor scale that is divided into somatic and cognitive factors.

Item 13 of the K-PSAS-16 (“Being mentally alert, active”), originally classified under the cognitive factor, demonstrated poor psychometric performance and was excluded in the final version. However, this result was not reported in previous studies involving translations into other languages, and may reflect differences unique to the Korean language and culture. We noted that while the remaining cognitive items were all negative items related to sleep, item 13 of the K-PSAS-16 contained positive connotations of the words “alert” and “active.” Given these findings, particular attention should be paid to the interpretation and translation of item 13 when assessing the psychometric properties of the PSAS in other languages in future research. In addition, a limitation of this study was that the sample size for each group did not exceed 100 participants [35]. Therefore, it is suggested that item 13 requires further analysis in future research to evaluate differential item functioning based on item response theory.

The K-PSAS-15 showed a significant correlation with other measurements assessing sleep quality, anxiety, and depression. The Swedish version of the PSAS was reported to be significantly correlated with sleep-related worries, sleep-related beliefs, anxiety, and depression [11]. The Turkish version of PSAS was also reported to have moderate to positive correlations with anxiety, depression, and sleep quality [33]. However, consistent with previous studies, the concurrent validity tests conducted in this study yielded several notable findings. First, among the four instruments examined, the K-PSAS-15 showed the weakest correlation with the HADS-D. Interestingly, the HADS-A, another subscale of the same instrument, yielded the strongest correlation, indicating a considerable discrepancy in concurrent validity between the two subscales. In the Swedish version of the PSAS, the correlation with the HADS-D was lower than with the HADS-A, which is consistent with the findings of the present study [11]. These results suggest that elevated anxiety levels may be associated with increased pre-sleep arousal, a relationship that warrants further investigation in future studies. Another notable finding is that the K-PSAS-15 somatic subscale showed lower correlation coefficients with all concurrent validity indicators compared to the K-PSAS-15 cognitive subscale. These findings are consistent with previous studies that have examined the validity of the PSAS. In the Portuguese version of the PSAS, the correlation coefficients between the PSAS-cognitive subscale and all sleep-related indices (e.g., sleep quality, sleep latency) were higher than those observed with the PSAS-somatic subscale [34]. Considering these results, the PSAS appears to be more appropriate for assessing cognitive arousal than somatic arousal.

Meanwhile, previous studies have reported on common problems with PSAS. Jansson-Fröjmark and his colleagues [11] were concerned that PSAS had low variance explained. In their study, the Swedish versions of PSAS-16, PSAS-13, and PSAS-14 reported explained variances of 47.1%, 48.5%, and 48.6%, respectively [11]. Several previous studies have shown that PSAS has a total explained variance ranging from 47.1% to 55.8% in two factors or ranging from 41.9% to 62.4% in three factors [11,36]. The explained variance is defined as the contribution of that factor to the total variation [37]. In social science, 50−60% of cumulative explained variance is known to be possible [33]. In this study, the explained variance of K-PSAS-16 and K-PSAS-15 were 49.34% and 52.54%, respectively, which were not particularly high but reasonably close to 50%. Therefore, K-PSAS-15 can be considered acceptable.

This study has several limitations that must be noted. Since this study analyzed secondary data, the participants were limited to e-cigarette or HTP users. Therefore, extending the research results to all adults in Korea may have limitations. Also, most of the participants in this study were between 31 and 50 years old, which may be because users of e-cigarette or HTP are often young adults. Therefore, we recommend that future studies consider stratified sampling to ensure balanced distribution of age groups. In addition, confirmatory factor analysis (CFA) was not conducted due to the nature of the secondary dataset. Although EFA was appropriate for the study’s objectives, future research should use independent samples to conduct CFA and further verify the factor structure of the K-PSAS-15. The use of a cross-sectional design limited the ability to assess test-retest reliability and responsiveness of the K-PSAS-15; these psychometric properties may be better evaluated through longitudinal studies.

## Conclusions

The findings of this study support the psychometric validity of the K-PSAS-15. EFA suggested a two-dimensional structure that aligns with the original PSAS, with items reflecting somatic and cognitive aspects of pre-sleep arousal. Item 13 was excluded due to poor psychometric performance, potentially reflecting cultural or linguistic factors. The K-PSAS-15 also showed significant correlations with measures of sleep quality, anxiety, and depression, especially with anxiety. These findings suggest that the K-PSAS-15 may serve as a culturally appropriate and reliable tool for assessing pre-sleep arousal in Korean adults who use e-cigarettes and HTPs. Its application could support the identification of individuals at risk for sleep disturbances within this population and contribute to the development of tailored interventions.

## Supporting information

S1 File PSAS row data. (XLSX)
